# Distribution grid impacts of electric vehicles: A California case study

**DOI:** 10.1016/j.isci.2021.103686

**Published:** 2021-12-28

**Authors:** Alan Jenn, Jake Highleyman

**Affiliations:** 1 Institute of Transportation Studies, University of California Davis, Davis, CA 95618, USA; 2 Cadmus Group, Oakland, CA 94612, USA

**Keywords:** Energy resources, Energy policy, Energy transportation, Energy storage, Energy systems

## Abstract

California has adopted a substantial number of electric vehicles over the last decade but there are many challenges associated with the electrification of vehicles, including how they interact with the electricity grid. We employ real-world feeder circuit level data in California from PG&E to measure the capacity of local feeders. We model the adoption of electric vehicles down to the census block and take advantage of real-world vehicle charging data to simulate the future loading on circuits throughout Northern California. In our highest adoption scenario of 6 million electric vehicles in California, we find that across PG&E's service territory, 443 circuits will require upgrades (nearly 20% of all circuits) and merely 88 of these feeders have planned upgrades in the future. The costs of these feeders are an essential part of a utility's planning process, and this work speaks to the importance of electric vehicles on local distribution networks.

## Highlights

• Electric vehicles will strain existing distribution infrastructure
• California's 2030 EV goals will require upgrades of 20% of distribution feeders in PG&E
• Utility forecasts underestimate need for planned upgrades

## Graphical abstract

**Figure:**
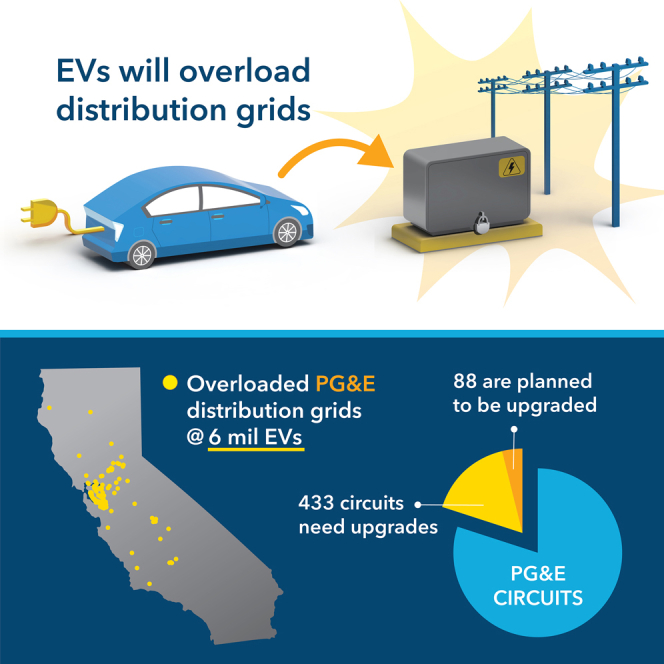

## Introduction

Since the passage of California's Global Warming Solutions Act of 2006 (Assembly Bill 32. California Global Warming Solutions Act of 2006. Chapter 488, September 27, 2006), requiring an 80% reduction of greenhouse gases (GHGs) below 1990 levels by 2050, the state of California has been a global leader in developing policies to combat climate change. The magnitude of carbon reduction means that all economic sectors in the state must undergo a transition to decrease their emission. As of 2018, transportation remains the largest contributor of GHGs in California, accounting for 41% of GHG emissions in the state (2000–2018 GHG Inventory, California Air Resources Board. https://ww2.arb.ca.gov/ghg-inventory-graphs). Over the last decade, a large suite of policies has been passed to reduce carbon emissions from the transportation sector. These include requirements to use cleaner fuels (Low Carbon Fuel Standards), mandate sales of zero emission vehicles (Zero Emission Vehicle [ZEV] and Advanced Clean Truck programs), and incentives for car buyers to purchase clean vehicles (Clean Vehicle Rebate Project, HOV Lane Access, and California Clean Fuel Reward) to name a few. These policies have helped to accelerate a transition away from traditional internal combustion engine (ICE) gasoline vehicles toward alternative technologies including full battery electric vehicles (BEVs), plug-in hybrid electric vehicles (PHEVs, which can operate both on a battery powered motor as well as a gasoline powered engine), and hydrogen fuel cell vehicles (FCVs).

The market for electric vehicles has developed rapidly over the last decade. The sales of electric vehicles have also grown substantially: a cumulative 800,000 vehicles have been sold within the state and in 2020 they accounted for nearly 9% of vehicle sales. As a result of their success, California has continued to push for aggressive adoption goals: in January 2018 former Governor Brown signed an executive order (Executive Order B-48-18, Governor Brown. January 2018) requiring 5 million ZEVs by 2030 and more recently Governor Newsom announced a ban on sales of new gasoline vehicles by 2035 (Executive Order N-79-20, Governor Newsom. September 2020). These measures will inevitably trickle down to California's regulatory agencies as they plan rules to support and ensure that the Governor's goals can be met over the next 15 years.

The electrification of the transportation sector poses several interesting challenges for the electricity sector in California (and outside the state as well, since California imports electricity from outside its borders). From an energy perspective, reaching the 2030 goal of 5 million electric vehicles could add on the order of 20 TWh annual electricity demand, an increase of about 10% of total electricity load in California. This increase comes amidst an overhaul of the state's electricity system as it strives to meet climate goals through regulation such as the Renewable Portfolio Standard (RPS) which requires a higher proportion of electricity generation from renewable energy sources such as solar or wind. From a power perspective, charging electric vehicles can represent a relatively higher power demand. Home chargers typically use Level 1 (about 1 kW) or Level 2 (about 6 kW) chargers, which is substantially higher than most applications in a residential setting. However, many public chargers employ DC fast chargers most of which are either 50 kW or 120 kW (primarily Tesla superchargers), though there are some applications of 350 kW extreme fast chargers for a small subset of passenger vehicles and for heavier duty applications. The energy and power demands related to electric vehicles pose an ongoing challenge for power providers and utilities across the state, especially given the rapid adoption of electric vehicles over the last decade and the pace of uptake required to meet California's aggressive climate goals. Nevertheless, the flexibility of vehicle charging demand also presents an opportunity for the electricity grid. Below, we provide an overview of electricity grid and electric vehicle integration studied in the literature, and provide a framework to understand our contributions to this area of study.

The uptake of electric vehicles coincides with increased utilization of renewable generation resources. Vehicle charging provides a unique complementarity to the intermittency of renewables due to their potential flexibility that can be exploited either through smart charging or vehicle-to-grid (V2G) services. This can be particularly beneficial for coupling with distributed resources to lower costs of renewable systems and of localized storage systems (Kandilet al., 2018; Klingler, 2018; Liu and Zhong, 2019). Several case studies of distributed level renewable integration impacts have been demonstrated in regions including Spain (Hernández et al., 2017) and Shanghai (Chen et al., 2020). In larger scale systems, similar studies have been conducted to take advantage of smart charging schemes to help integrate with larger volumes of renewable generation facilities even in the presence of uncertainty in generation due to intermittency (Laurischkat and Jandt, 2018; Ata et al., 2019; Mehrjerdi and Rakhshani, 2019; Langenmayr et al., 2020; Rezaeiet al., 2020). Studies have investigated these integrated renewable, storage, and EV systems both on hypothetical circuits (Rahbariet al., 2017; Morshed et al., 2018) and in real locations such as New York City (Freeman et al., 2017) and regions within China (Sun, 2021). Simply from the perspective of renewables and storage integration, these studies demonstrate a substantial theoretical cost savings and provide valuable insight into the operational aspects of such systems—both on a distributed and larger-scale systems.

In addition to the cost savings by coupled operation, taking advantage of charging load flexibility also provides explicit economic benefits through participation in certain markets available to participants in markets related to the electricity grid. This may include frequency reserve markets (Borne et al., 2018), energy and reserve markets (Carriónet al., 2019a; Wu and Lin, 2021), and real-time trading services (Meisel and Merfeld, 2018). Furthermore, widespread adoption of V2G services opens the door to a large number of value streams, including bill management, resource adequacy, network deferral, energy arbitrage, and spinning reserves (in addition to the aforementioned markets) (Thompson and Perez, 2020). Many studies have also examined how flexibility in vehicle charging load can be taken advantage of through a variety of demand-side mechanisms. These programs include demand response participation, with electric vehicles providing a large potential benefit due to their relatively large power draw (Aliasghariet al., 2018; Jabariet al., 2019). Given the complexity and market requirements of many of these services, it is unlikely that individual customers would be able to capture these benefits; nevertheless they represent a substantial economic opportunity that could be captured in the future. Nevertheless, studies have provided insight into how consumers should be compensated to entice them to participate in grid programs (Das et al., 2020).

As electric vehicle demand increases so too does the corresponding load requirements for charging the vehicles. The expansion of power generation and transmission capacity is therefore a critical question to address whether the electricity grid can handle the increase in electricity load. This issue has been examined in capacity investment models (Carrión et al., 2019b) and for transmission systems (Gunkelet al., 2020). Generally, studies have indicated that the greater amount of load flexibility is taken advantage of, the lower the capacity requirements are for wholesale generation and transmission within the electricity system. However, in most examinations of future scenarios, the required capacity increases needed to meet EV charging loads are still relatively small (Taljegardet al., 2019; Jennet al., 2020).

While the wholesale elements of the electricity grid will likely not be extremely stressed by charging loads in the near future, the local distribution infrastructure faces a different set of constraints. The work conducted in this study is most closely related to capacity issues at this scale. Most grid integration issues have been at larger wholesale generation and transmission levels, though we outline a limited body of literature on interactions between EVs and the distribution grid below. Even before the availability of electric vehicles on the market, Clement-Nyns et al. examined hypothetical impacts on the distribution grid in terms of power loss and voltage deviations by employing a 34-node test feeder. The results of their analysis reveal that coordinating charging can significantly reduce power losses and improve power quality by flattening peak power events caused by vehicle charging (Clement-Nyns et al., 2010).

While the benefits of coordinated smart charging are readily apparent for distribution grids, they also face many barriers including practical attributes of EV flexibility, ability to observe and provide feedback to the vehicle from the grid (and through smart meters), limitations of EV supply equipment to access the electricity network, EV communication standards, and a host of regulation and market barriers. A combination of technical and policy solutions are required to overcome these issues and allow for load flexibility to be realized (Knezovićet al., 2017; Gonzalez Venegas et al., 2021). Several case studies have estimated benefits in cost savings to transformer capacities when comparing smart charging schemes to uncontrolled EV charging scenarios: cost savings as high as 32% in the Netherlands (Brinkelet al., 2020) and a reduction in reinforcement of the distribution grid from 28% down to 9% in Great Britain (Crozier et al., 2020). However, these models employ simplistic assumptions for charging behavior—an important nuance that can shift charging loads to problematic coincident peaks depending on the assumptions being made. Muratori most closely resembles the work conducted in this study; however, the impacts on the distribution network are based on a small case study of households and limited in scope to average attributes of distribution networks (Muratori, 2018).

This study presents the first empirical evaluation of existing feeder infrastructure and the corresponding overload impacts on the electricity distribution system when adding electric vehicle charging load. As the number of electric vehicles on the road continue to grow overtime, our study attempts to quantify the impacts of their charging loads by determining which distribution network feeders will exceed their rated capacity thresholds. These impacts on distribution feeders within Pacific Gas & Electric's (PG&E) utility region are determined in the following steps:

1. Simulation of electric vehicle charging loads across several scenarios of vehicle adoption
2. Application of additional loads to existing loads to each individual feeder within PG&E
3. Evaluate the status of feeders on the basis exceedance of capacity thresholds

Our work takes advantage of highly detailed data on distribution networks from open-source utility databases in California that provides individual transformer capacities and historical load information, a substantial departure from modeled assets in previous studies. While the availability of data limits our analysis to PG&E territory, the findings from our work will have important implications for any distribution networks that experience substantial electric vehicle adoption. Additionally, the work in this study employs state-of-the-art empirical data on charging profiles which often differ substantially from how many studies assume drivers charge their vehicles (e.g. charging as soon as they arrive home from work).

### Data description

#### Integration Capacity Analysis (ICA) data and maps

**Figure 1:**
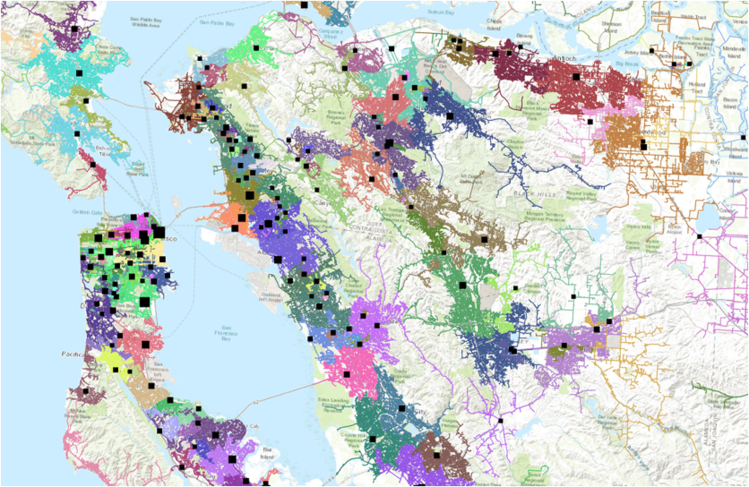
Integration Capacity Analysis map for Pacific Gas & Electric (PG&E) utility's service in Northern California (Greater Bay Area and surrounding regions) Black squares represent substations.

In 2016, the California Public Utilities Commission (CPUC) established a working group as part of their Distribution Resource Plans that required utilities to develop a publicly available dataset called the Integration Capacity Analysis (ICA) map. These maps provide access to contractors and developers to plan siting and installation of distributed energy resources (DERs) including solar and storage infrastructure. The data contain information on transformer capacity at the substation level or below, line capacities down to the circuit level, and load profiles all at high spatial resolution. We show part of the coverage of investor-owned utility Pacific Gas & Electric's (PG&E) distribution network in the Greater Bay Area of California (Figure 1).

Across PG&E's network, we are able to observe 2,135 feeder lines spread across four major regions of California: the Bay Area, Central Coast, Central Valley, and Northern California (north of the Bay Area and west of the Sierra Nevada mountain range). Specific feeder information is contained within the Grid Needs Assessment (GNA) dataset as part of the ICA. The GNA contains load profile information for each feeder within PG&E's network. We observe the highest and lowest hourly load on each feeder circuit for each month of the year, as well as projections for changes in future peak load due to energy efficiency improvements and rooftop PV solar installations. As we are primarily concerned with exceeding capacity thresholds for feeders, we will focus on the highest load periods in the month. Note that there is a large diversity of both hourly load shapes across the day between feeders, as well as a large diversity in seasonal changes in peak load between feeders as well. Some feeder lines have fairly constant peak loads throughout the year (minimal seasonal variation). While peak loads do differ between circuits, the distribution of peak load across the entire day is relatively constant. Across the 2,135 feeders, the lowest (first percentile) load reaches 268 kW while the highest (99^th^ percentile) load reaches 15.8 MW (see Figure S1 in the SI).

#### Electric vehicle miles traveled and residential charging behavior data

Much of the existing literature on electric vehicle charging employs modeling charging behavior based on large-scale travel diaries of internal combustion engine (ICE) gasoline vehicles. However, there are important distinctions between travel behavior between vehicle technologies (particularly for early adopters of EVs) as well as observed charging patterns compared to modeled patterns. In this study, we employ data from a multiyear study by the University of California, Davis for the California Air Resources Board to monitor EV usage in California (Raghavan and Tal, 2020; Tal et al., 2020). The study employed data loggers that collected second-by-second vehicle usage data from current EV owners in California. Participants for the logger study are initially filtered from an online survey which recruits randomly based on a combination of enrollment for California's CVRP incentive and from the Department of Motor Vehicle (DMV) records using stratified random proportionate sampling. The stratification is across five of the largest utilities in California: PG&E, San Diego Gas & Electric (SDGE), Southern California Edison (SCE), Sacramento Municipal Utility District (SMUD), and Los Angeles Department of Water and Power (LADWP). Despite coverage through multiple utility areas, we find that charging patterns throughout different service territories do not vary substantially and therefore a full sample can be used to represent the charging profiles within PG&E.

**Figure 2:**
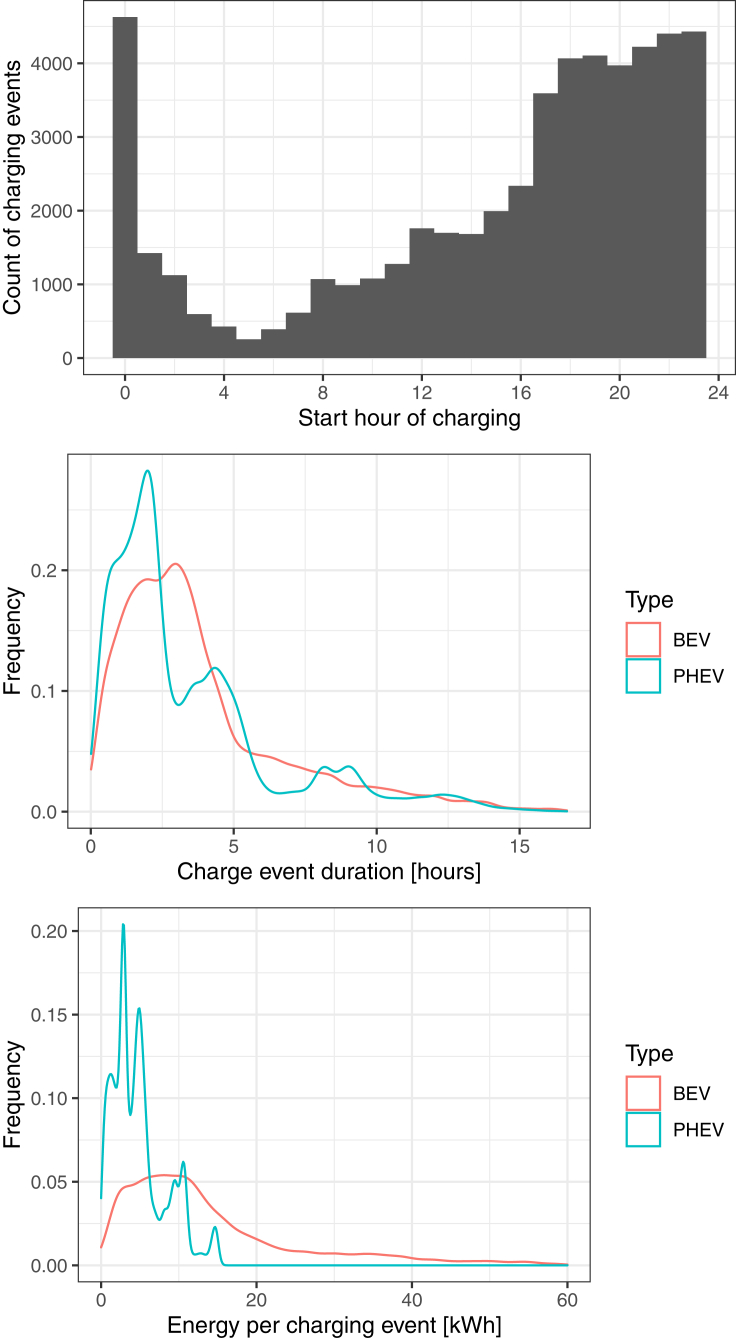
Summary of distributions for residential charging events from logged EVs in California Distributions are provided for the starting hour of charging events (top), duration of each charge event broken down by BEV and PHEV (middle), and the energy dispensed per charging event broken down by BEV and PHEV (bottom).

The current dataset logs a total of 233 unique EVs consisting of 6 of the most popular vehicle models over the course of a full year. Since we employ a separate comprehensive dataset for public charging events, we employ only residential charging events from the logger dataset. The data culminate to a total of 52,146 separate charging events with a total of 2,991,064 miles traveled. We show several attributes of charging behavior in Figure 2. In terms of timing of charging, most EV drivers tend to begin charging their vehicles in the evening starting around 4 or 5 PM extending through midnight. We observe a large peak starting at midnight due to automatic charging timer delays in vehicles that allow users to delay the start of their charging until a preset time (many vehicles have a default setting at midnight). The average length of a single residential charging session is about three and a half hours, though there is a distinct difference in distribution between PHEVs and BEVs in Figure 2. This is likely due to the difference in battery sizes between PHEVs (which will tend to have smaller batteries) and BEVs. This is also reflected in the amount of electricity dispensed per charging event, with full BEVs averaging 14 kWh compared to PHEVs averaging 4.8 kWh. One of the most notable differences between many modeling approaches in existing studies compared to the empirical data is that vehicles are often assumed to charge every day, as well as to charge until the vehicle is fully charged. However, in the empirical data, we find that neither of these assumptions holds true and most EV owners intermittently charge their vehicle throughout the week and that many charge events do not fully charge the battery a 100% state-of-charge (SOC). These assumptions are particularly important in simulating the electricity load on electricity infrastructure.

#### Public and work charging behavior data

While charging EVs at public charging stations accounts for a relatively small proportion of overall charging events (Lee et al., 2020), a substantial amount of the charging that occurs at public infrastructure is at DC fast charging stations which charge vehicles at significantly higher power compared to residential charging. Most charging at home is typically at Level 1 or Level 2, which usually corresponds to 1 kW for the former and around 6–7 kW for the latter. In comparison, DC fast charging stations are at least 50 kW with newer stations at 100 kW and Tesla superchargers at upwards of 120–150 kW. Extreme fast chargers are even being developed to charge at rates of 350 kW (Jennet al., 2020). Despite the lower number of events, the higher power requirements can easily exceed the local line or transformer capacities of electricity distribution infrastructure—a single car charging at 50 kW is equivalent to the power draw of fifty vehicles simultaneously charging at their home on a single circuit.

To simulate future public charging events, we employ a massive dataset consisting of nearly 6 million charging events from 2014 through 2019 in California. The data contain comprehensive records from several charging network providers (including EVGo, Chargepoint, and Electrify America). Similar to the eVMT dataset we use for characterizing residential charging behavior, each record contains information on the start and end time of the charging event, the total amount of power charged, and the location/characteristics of the charging station. We observe a substantially different charging behavior for public charging in comparison to residential charging with a uniform peak starting around 3 PM till around midnight with a relatively smaller discrepancy between off-peak hours compared to residential charging. Additionally, the average electricity dispensed at public chargers is lower in quantity compared to residential charging at 12.6 kWh per event, despite the higher power available in public chargers.

The charging behaviors are then applied to the distribution of forecasted adoption of EVs throughout California (see Figure S2 in the SI). The remainder of our study presents our results describing our EV charging load simulation, the diversity of load impacts through case studies of individual feeders, and an overview of aggregate impacts on distribution networks throughout California; lastly, we provide a discussion of the implications of our findings and recommendations for policy and future research.

## Results

We find that the growth of electric vehicles in California will inevitably lead to stresses on the electricity distribution infrastructure due to the additional electricity demand from charging these vehicles. Our results demonstrate the magnitude of these impacts across different scenarios of electric vehicle adoption that may correspond to levels of penetration over the next decade. The findings are organized into three major sections: 1) results from the EV charging load simulation, 2) investigation of the diversity of load impacts on individual feeder circuits, and 3) an overview of aggregate impacts on feeders across PG&E's service territory. All results from our work are based on impacts of feeders within PG&E regions.

### Charging load simulation

**Figure 3:**
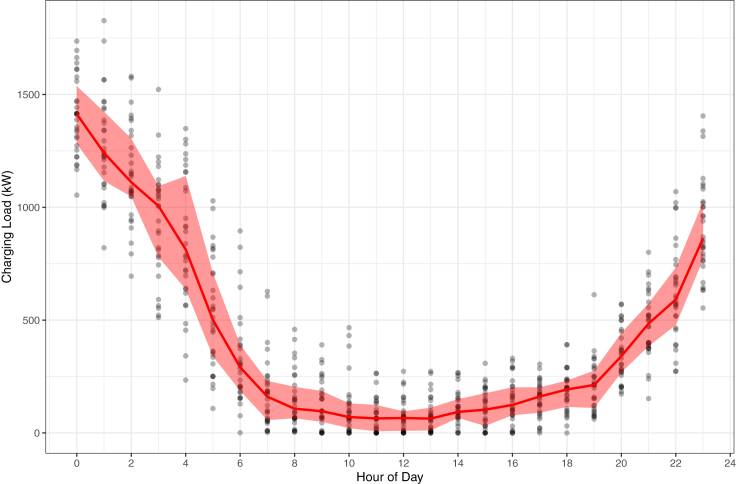
Simulated aggregate electric vehicle load of 1000 full BEVs with a ratio of 84% long-range vehicles (>100 miles) and 16% short-range (<100 miles) over the course of a full day with 30 trials to represent the variation seen in a single month.

A sample of simulated vehicle charging outcomes can be seen in Figure 3. While the red line shows the median load value for 1,000 vehicles over the course of a day, we observe a substantial amount of variation in load (+/− 30% of the median load) due to the variability of vehicles deciding to charge on a given hour as well as the speed of charging from the bootstrapped simulation. It should be noted that while there are 1,000 vehicles with a peak load of 1.5 MW, this does not necessarily mean that the majority of charging is on Level 1 chargers, but also reflects the fact that not all of the vehicles will charge every single day, thus leading to lower total load than if vehicles were to choose to charge to full capacity every day (as is assumed in most models in the literature). From around 6 AM through 6 PM, we observe a low amount of charging load. Despite higher power events taking place during the day from DC fast charging at public stations, the proportion of charging events that happens during this time period is relatively small compared to residential charging. However, starting around 7 PM, charging load demand rapidly grows up until midnight before beginning to decrease in the early morning. The peak load almost always begins at midnight due to timer settings within the vehicle that are programmed to start at 12 AM. While we sample electric vehicles across the state, we do not find substantial differences in charging patterns between utility territories and therefore apply a bootstrap of our full sample to simulate charging load within PG&E.

When comparing the shape of the charging demand load to baseload electricity demand, the peaks are not coincident. However, peak baseload often occurs in the early evening in certain regions and seasons which coincides with the time that charging load demand begins to increase for the day. Additionally, we note that charging demand is lowest during the day, which is nearly the opposite profile of renewable solar generation. While this issue is not particularly relevant when considering distribution infrastructure impacts for utility-scale solar, residential rooftop and local solar generation can have a mitigating effect on transformers and feeder lines if utilized correctly. This points to substantial opportunity for managed charging events, even with smart charging (as opposed to vehicle-to-grid), by load shifting many of the peak events can be substantially ameliorated if not outright eliminated—thus reducing the need for transformer and other distribution infrastructure upgrades.

### Load impacts on individual feeders

**Figure 4:**
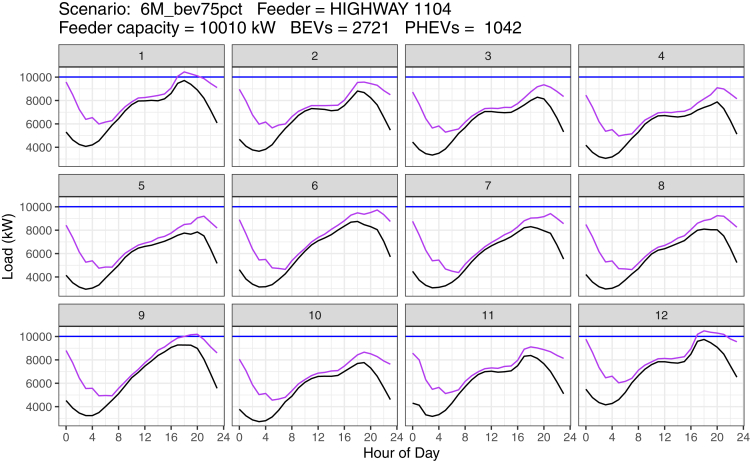
Example of the peak load day event in each month of the year for a single feeder circuit with a capacity of about 10 MW In a scenario of 6 million electric vehicles, we forecast 3,763 vehicles adding charging load to the feeder. The threshold capacity of this feeder is exceeded in three months of the year (January, September, and December) and is dependent on the peaking of baseload. Black line: Baseload. Purple line: Baseload + EV Load. Blue line: Feeder Capacity.

Unlike many studies of grid management at the wholesale generation and transmission level, there is a massive diversity of infrastructure impacts at the distribution level. Our work examines the effect of electric vehicle adoption throughout California, ranging in scenarios from 1 million vehicles (slightly beyond existing EV volumes in 2021) up to 6 million vehicles (approximately 20% above California's stated goals of reaching 5 million EVs by 2030). Owing to the variation in the shape of baseload demand, the effect of additional charging load on reaching capacity thresholds differs substantially between feeders. It is important to note that the additional charging load is not a uniform effect across the year. In Figure 4, we show an example of a particular PG&E feeder with approximately 3,800 EVs added; its capacity threshold is exceeded on peak load days in only 3 months of the year. In many other months, the headroom (the amount of capacity between the baseload and feeder capacity) can be decreased by as much as 60%. Analysis over a full year provides insight into the frequency and intensity with which threshold capacities are reached, as these events increase about those two attributes, the faster the hardware begins to degrade. Across all feeders in our analysis, we find that the maximum exceedance of load capacity reaches over 300% of a feeder's threshold (in Sonoma County). Additionally, EV loads may increase the amount of time that threshold capacities are exceeded—extended periods of time in exceedance can accelerate the degradation of infrastructure (for example, decreasing transformers' ability to passively cool). In extreme cases, we identify instances of loads exceeding capacity as long as 22 h (in West Sacramento).

Ideally, we would run our simulation and add charging load across a load on the feeder across the full year. Unfortunately, the data are not readily available, but we are able to observe seasonal effects on peak load events, allowing us to be confident on results for feeders that do not reach their thresholds.

Feeder threshold capacity being reached is one example of an outcome from adding charging load to the distribution system. Generally, additional charging load can lead to two other possibilities: 1) the load would fail to reach the capacity threshold of the feeder or 2) the feeder's threshold is already exceeded during peak events and additional charging load simply accelerates hardware degradation in the feeder network. In the former case, the existing distribution network can handle the additional load from charging EVs, though the instances of this case decrease as adoption of EVs increase over time and corresponding charging loads increase throughout the state. These issues are discussed in the aggregate in the following section.

### Aggregate impacts of charging load on distribution systems

**Figure 5:**
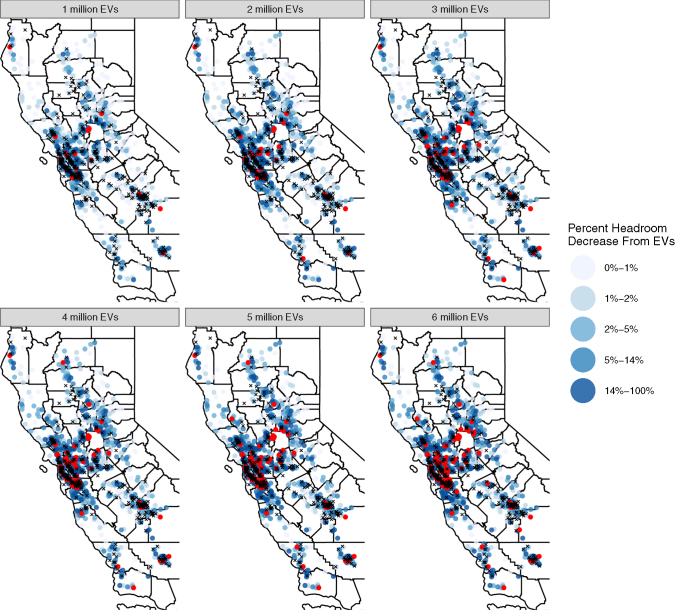
Decrease in headroom (spare capacity) of feeder circuits (represented as points at the centroid of the network) during peak load events due to electric vehicle charging Red dots indicate feeder circuits that would exceed their rated capacities due to vehicle charging and black X's indicate feeders whose capacities are exceeded during peak load regardless of EV charging.

Ultimately, the importance of EV charging load depends on how frequently the additional electricity demand stresses the distribution network (which we demonstrate in the previous section as when feeder capacity limits are reached). In Figure 5, we can view how feeder circuits (represented at the centroid of their respective networks as points) become increasingly stressed as more electric vehicles are adopted. The percentage of remaining headroom decreases on average, particularly in regions with large forecasts of EV adoption. Perhaps most striking is the increasing density of circuits whose threshold capacity is met (shown as red points) as more electric vehicles are introduced onto distribution networks throughout the state. While many feeders can retain loads below their thresholds, our simulation of vehicle charging reveals a non-negligible amount of required upgrades to the distribution system to support the increased demand. We also note that the final vehicle adoption scenario represents only the upgrades likely required over the next decade. If California were to meet its decarbonization goals by 2045, this would probably require upgrades across the entire distribution network.

**Figure 6:**
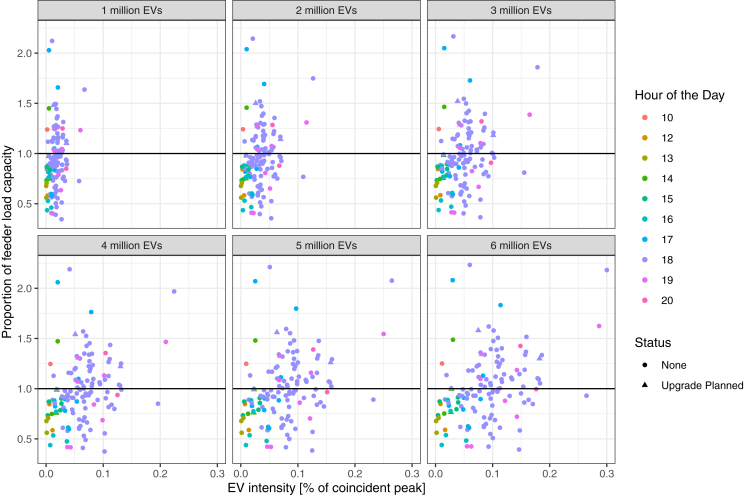
Status of feeders in the San Francisco Peninsula (spanning from the south of San Francisco City to the city of Santa Clara) showing the peak load relative to the feeder load capacity and the proportion of the peak load coming from electric vehicle charging The results span across 6 scenarios of EV adoption. Some feeder lines are planned to be upgraded (shown as a triangular shape), though the majority of feeders exceeding the threshold capacity are not planning to be upgraded.

**Figure 7:**
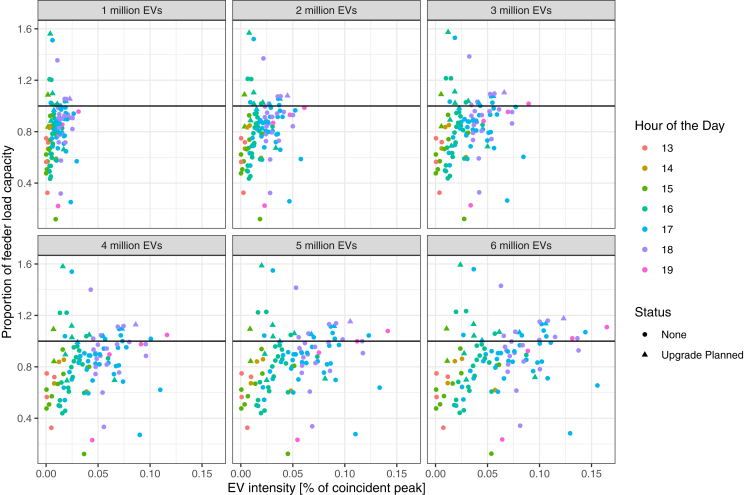
Status of feeders in the city of San Jose showing the peak load relative to the feeder load capacity and the proportion of the peak load coming from electric vehicle charging The results span across 6 scenarios of EV adoption. Some feeder lines are planned to be upgraded (shown as a triangular shape), though the majority of feeders exceeding the threshold capacity are not planning to be upgraded.

In PG&Es Grid Needs Assessment, the utility provides information on feeders that have planned capacity upgrades in their distribution network. We find that the necessary upgrades are inadequate to meet the increased load demand from EV charging. Of feeders that experience charging load demand beyond their threshold capacity, only a fraction of these feeders has planned upgrades (see Figures 6 and 7, note the difference in axis scales). In the 6 million vehicle scenario, there are a total of 443 feeders exceeding their capacity threshold, yet only 88 of these feeders will have upgrades that will allow them to feasibly operate in the long-term. As the number of electric vehicles increases, we observe that the proportion of the coincident peak attributable to vehicle grows continuously. For example, as seen in Figure 6, within the San Francisco Peninsula and 1 million electric vehicles in California, the majority of feeders contribute less than 5% (with the highest around 7%) of peak load. By the time 6 million vehicles are adopted in California, charging demand is responsible for upwards of 20% of peak load (and reaching as high as 30%). In San Jose, Figure 7, a similar pattern can be observed: under a 1 million EV adoption scenario the contribution to peak load is only 5% or lower, while the proportion of peak load reaches as high as 15% once adoption levels reach 6 million EVs in California.

We are also able to provide some insight into the relative timing of peak loads. This information is an essential parameter for operators seeking to identify opportunities for flexibility and how load shifting of charging demand may help to mitigate increasing the intensity of peaks across feeders in specific regions. In Figure 6, on the San Francisco Peninsula, we observe that most of the peak events at on the feeder occur at approximately 6 PM. However, these peaks may differ by region as we observe in San Jose in Figure 7, there is a much wider distribution of peak times beginning around 3 PM through around 6 PM—though the peaks with highest EV intensity tend to be in the latter hours closer to or at 6 PM.

Across PG&E's distribution network, the demand load from electric vehicle charging clearly has a large effect on peaking load beyond the capacity threshold of feeder lines. Additionally, the majority of these events do not have planned hardware upgrades, which may result in widespread accelerated degradation of distribution networks in Northern California.

## Discussion

The state of California has some of the most aggressive climate policies in the world through their host of legislated and regulated policies (Cap and Trade, Low Carbon Fuel Standards, Renewable Portfolio Standards, and the Zero Emission Vehicle mandate to name a few). The contribution of the transportation sector to the state's emissions has thus motivated many of California's regulatory agencies to target transportation for decarbonization. The trend in electric vehicle adoption over the last decade has reflected many of the successes of these policies to push the technology to be accepted by more of the population every year. However, a broad shift toward electric vehicles may lead to substantial impacts on the electricity grid.

Our work focuses on the distribution infrastructure, primarily consisting of the grid network between local substations and the end customers. We examine several scenarios of electric vehicle adoption in California, up to 6 million EVs, using an existing EV adoption model (EV Toolbox, see Supplemental information) which allows us to locate electric vehicles throughout the state at a high-level of spatial resolution. We then simulate vehicle charging demand and perform a case study of Northern California utility Pacific Gas & Electric's distribution system. Owing to the wide variety of baseload demand and available capacity on local feeder lines, the effect of adding electric vehicle charging load differs substantially between feeders on PG&E's network and over different periods of the year. The impact on feeders can be negligible for instances where the additional electricity load does not approach the threshold capacity of feeder lines, but when loads exceed peak capacity of existing hardware this may lead to accelerated degradation of equipment on the network. Degradation can be accelerated based on the relative intensity of peaks beyond capacity as well as by the length of time that capacity is surpassed. Across the population of feeders on PG&E's network, we observe both instances with peak intensities reaching 300% of capacity as well as feeders whose thresholds are surpassed for over 22 h within a single day. In aggregate, issues related to capacity availability become increasingly frequent at higher volumes of electric vehicles. In our scenario of 6 million EVs adopted across California, over one-fifth of feeders have their capacities exceeded due to charging events—only a fraction of these feeders are planned to be upgraded by the utility over the next few years. The impact of charging load on the distribution network will likely have large impacts on utilities throughout California over the next decade and regulatory agencies such as the California Public Utilities Commission and the California Energy Commission will likely play a critical role in assisting utilities to mitigate these issues.

### Limitations of the study

Our work suffers from several shortcomings in data availability and assumptions that we or others may improve upon in future work. Firstly, our work only examines peak hourly load for each month whereas a true indication of frequency of load exceeding capacity would require hourly baseload data across the entire year. Secondly, patterns of charging have slowly evolved over time—the shape of charging load has shifted over the last decade as different consumers adopt the technology and as battery sizes increase over time. While we base our simulations on empirical data, future charging load may differ from patterns that we observe today. Future work can augment our analysis by including forecasts of the electrification of medium and heavy-duty vehicles that will likely have an even larger impact on distribution systems due to the high-power requirements to charge those vehicles. Additionally, work examining the potential to mitigate the impacts of vehicle charging on distribution systems by taking advantage of the inherent flexibility of vehicle charging may prove to be critically important to reducing some of the costs associated with EV adoption on the electricity grid. Lastly, we are limited by the attribute information provided through ICA on the physical characteristics of the feeder hardware. Additional data on voltage and current ratings of feeders could augment the analysis and provide better insight into the degradation of the distribution system.

## STAR★Methods

### Key resources table

**Table:** 

REAGENT or RESOURCE	SOURCE	IDENTIFIER
**Deposited data**		

Distribution network data	Integrated Capacity Analysis Maps	https://www.pge.com/en_US/for-our-business-partners/distribution-resource-planning/distribution-resource-planning-data-portal.page?ctx=large-business
Electric vehicle adoption projections	EV Toolbox	https://phev.ucdavis.edu/project/uc-davis-gis-ev-planning-toolbox-for-mpos/

**Software and algorithms**		

R 4.1.2	The R Project for Statistical Computing	https://www.r-project.org/

### Resource availability

#### Lead contact

Further information and requests for resources should be directed to and will be fulfilled by the lead contact, Alan Jenn (ajenn@ucdavis.edu)

#### Materials availability

This study did not generate new unique materials.

### Methods details

#### Characterizing transformer and feeder circuit capacities

We combine the Grid Needs Assessment capacity data with the feeder load data to assess a baseline of load demand for feeder overloading. In Figure S1 in the SI, we show the range of feeder capacities as well as their corresponding peak load across the year within PG&E's service territory. Note that the capacities are not physical thresholds, but rather are thresholds set to maximize operational longevity across the circuit and for infrastructure equipment on the feeder line. We observe that approximately 15% of feeders in the data will exceed their capacity threshold at least once across a full year (though to limitations in the availability of hourly data across the entire year, we are not able to observe how often peak load exceeds the capacity). These events will accelerate the degradation of distribution infrastructure, particularly the more often they occur. Our study simulates EV load across a range of forecast assumptions to observe how additional load from charging events, particularly as California advances the technology to meet their climate change goals, will increase stress on distribution infrastructure as peak loads are pushed upwards toward feeder line capacity limits.

#### Electric vehicle adoption using EVtoolbox and load simulation

We employ an electric vehicle adoption forecasting tool called EV Toolbox used to generate official estimates for California Environmental Protection Agency's (CalEPA) planning forecasts for statewide transport decarbonization (Tal et al., 2020; Brown et al., 2021). The tool adopts ZEVs through a series of assumptions: electrification occurs at the household level one vehicle at a time, income and housing type are the primary explanatory variables (wealthier households and households with the possibility of installing charging infrastructure), relative proportions of household types and vehicle ownership remains static, and the stock of vehicles stays relatively constant. More details on the adoption model can be found in previous studies employing the EV Toolbox model—our work mainly employs outputs as scenarios of adoption.

Our work makes no assumptions on the speed with which EVs are adopted but instead takes the spatial distribution of the vehicles from the EV Toolbox as a given. Rather than approximate a volume of EVs in a certain year, we simply examine scenarios of EV penetration. For example, Figures S2 in the SI shows the spatial distribution by census block groups of electric vehicles in a scenario with 6 million EVs with a split of 75% BEVs and 25% PHEVs. It is uncertain when this scenario would occur, but if California were to keep pace with its stated goal of 5 million EVs on the road in 2030, it is highly likely that the scenario could be achieved before 2035. Note that when dividing the count of EVs registered in each region by quantile, the bottom 80^th^ percentile has on the order of several hundred vehicles per census block group (which corresponds to the average population per block group between 600 and 3,000) though the upper percentiles include as high as 20,000 EVs within a single block group. We ultimately examine scenarios of EV adoption in increments of 1 million vehicles adopted up to 6 million EVs.

Additionally, in the eVMT data we observe differences in charging behavior between electric vehicle models that are primarily characterized by the size of battery and corresponding range of the vehicle. As a result, we also separate the forecasted electric vehicles are divided into long-range and short-range BEVsand PHEVs. This division is conducted by examining empirical data on EV sales in California—employing data from the Clean Vehicles Rebate Program, we find that long-range BEVs account for 84% of full battery electric vehicles while long-range PHEVs account for 58% of plug-in hybrids. Charging loads are bootstrapped from the eVMT data, with a separate distribution for each short/long-range PHEV and BEV vehicle types respectively. The bootstrapping procedure is conducted by generating separate distributions of hourly charging probabilities for the vehicle types and charging speeds from our observed data. We then probabilistically sample each hour of the day based on these distributions to determine whether a vehicle is charging in each hour (for every vehicle type separately). The individual charging profiles are then summed to generate an aggregate charging load profile within each census block group. The empirical data contain information on the timing and speed (power) of charging, allowing us to accurately represent the charging patterns of existing vehicles for a simulated volume of new electric vehicles in California.

#### Spatially connecting distribution infrastructure to EV adoption

One of the challenges of connecting electric vehicle charging events to the grid is that the electric vehicle adoption locations from EV Toolbox operate in different spatial regions than the distribution network. There are two issues of misalignment: a single census block group can contain multiple feeder circuits and a feeder may also extend across multiple block groups. To solve this issue, we re-allocated EVs from census block groups down to census blocks based on the proportion of populations within the block for each block group. Once vehicles are allocated to census blocks, we perform a spatial intersect between the blocks and the distribution network, a procedure which assigns a block to a specific feeder line. When attempting this procedure with a block group, multiple feeders would be assigned to a single group. However, since census blocks are small enough to avoid most of the misalignment issues, we can spatially match feeder circuits to individual census blocks—thus allowing us to assign the forecasted EVs within census block groups to each of the feeders across PG&E's service area.

## Data Availability

Datasets for distribution network information and electric vehicle projections are available in the key resources table. Any additional information required to reanalyze the data reported in this paper is available from the lead contact upon request. All original code has been deposited on GitHub from https://github.com/headisbagent/EV_DistributionGrid_Analysis. DOI for initial release: https://doi.org/10.5281/zenodo.5773655.

## References

[bib1] Aliasghari P., Mohammadi-Ivatloo B., Alipour M., Abapour M., Zare K. (2018). Optimal scheduling of plug-in electric vehicles and renewable micro-grid in energy and reserve markets considering demand response program. J. Clean. Prod..

[bib2] Ata M., Erenoğlu A.K., Şengör İ., Erdinç O., Taşcıkaraoğlu A., Catalão J.P. (2019). Optimal operation of a multi-energy system considering renewable energy sources stochasticity and impacts of electric vehicles. Energy.

[bib3] Borne O., Perez Y., Petit M. (2018). Market integration or bids granularity to enhance flexibility provision by batteries of electric vehicles. Energy Pol..

[bib4] Brinkel N.B.G., Schram W.L., AlSkaif T.A., Lampropoulos I., Van Sark W.G.J.H.M. (2020). Should we reinforce the grid? Cost and emission optimization of electric vehicle charging under different transformer limits. Appl. Energy.

[bib5] Brown A., Sperling D., Austin B., DeShazo J.R., Fulton L., Lipman T., Murphy C., Saphores J.D., Tal G. (2021).

[bib6] Carrión M., Domínguez R., Cañas-Carretón M., Zárate-Miñano R. (2019). Scheduling isolated power systems considering electric vehicles and primary frequency response. Energy.

[bib7] Carrión M., Domínguez R., Zárate-Miñano R. (2019). Influence of the controllability of electric vehicles on generation and storage capacity expansion decisions. Energy.

[bib8] Chen J., Zhang Y., Li X., Sun B., Liao Q., Tao Y., Wang Z. (2020). Strategic integration of vehicle-to-home system with home distributed photovoltaic power generation in Shanghai. Appl. Energy.

[bib9] Clement-Nyns K., Haesen E., Driesen J. (2010). The impact of charging plug-in hybrid electric vehicles on residential distribution transformers. IEEE Trans. Power Syst..

[bib10] Crozier C., Morstyn T., McCulloch M. (2020). The opportunity for smart charging to mitigate the impact of electric vehicles on transmission and distribution systems. Appl. Energy.

[bib11] Das R., Wang Y., Putrus G., Kotter R., Marzband M., Herteleer B., Warmerdam J. (2020). Multi-objective techno-economic-environmental optimisation of electric vehicle for energy services. Appl. Energy.

[bib12] Freeman G.M., Drennen T.E., White A.D. (2017). Can parked cars and carbon taxes create a profit? The economics of vehicle-to-grid energy storage for peak reduction. Energy Policy.

[bib13] Gonzalez Venegas F., Petit M., Perez Y. (2021). Active integration of electric vehicles into distribution grids: barriers and frameworks for flexibility services. Renew. Sustain. Energy Rev..

[bib14] Gunkel P.A., Bergaentzlé C., Jensen I.G., Scheller F. (2020). From passive to active: flexibility from electric vehicles in the context of transmission system development. Appl. Energy.

[bib15] Hernández J.C., Ruiz-Rodriguez F.J., Jurado F. (2017). Modelling and assessment of the combined technical impact of electric vehicles and photovoltaic generation in radial distribution systems. Energy.

[bib16] Jabari F., Jabari H., Mohammadi-ivatloo B., Ghafouri J. (2019). Optimal short-term coordination of water-heat-power nexus incorporating plug-in electric vehicles and real-time demand response programs. Energy.

[bib17] Jenn A., Clark-Sutton K., Gallaher M., Petrusa J. (2020). Environmental impacts of extreme fast charging. Environ. Res. Lett..

[bib18] Kandil S.M., Farag H.E., Shaaban M.F., El-Sharafy M.Z. (2018). A combined resource allocation framework for PEVs charging stations, renewable energy resources and distributed energy storage systems. Energy.

[bib20] Klingler A.L. (2018). The effect of electric vehicles and heat pumps on the market potential of PV + battery systems. Energy.

[bib21] Knezović K., Marinelli M., Zecchino A., Andersen P.B., Traeholt C. (2017). Supporting involvement of electric vehicles in distribution grids: lowering the barriers for a proactive integration. Energy.

[bib22] Langenmayr U., Wang W., Jochem P. (2020). Unit commitment of photovoltaic-battery systems: an advanced approach considering uncertainties from load, electric vehicles, and photovoltaic. Appl. Energy.

[bib23] Laurischkat K., Jandt D. (2018). Techno-economic analysis of sustainable mobility and energy solutions consisting of electric vehicles, photovoltaic systems and battery storages. J. Clean. Prod..

[bib24] Lee J.H., Chakraborty D., Hardman S.J., Tal G. (2020). Exploring electric vehicle charging patterns: mixed usage of charging infrastructure. Transport. Res. D: Transport Environ..

[bib25] Liu J., Zhong C. (2019). An economic evaluation of the coordination between electric vehicle storage and distributed renewable energy. Energy.

[bib26] Mehrjerdi H., Rakhshani E. (2019). Vehicle-to-grid technology for cost reduction and uncertainty management integrated with solar power. J. Clean. Prod..

[bib27] Meisel S., Merfeld T. (2018). Economic incentives for the adoption of electric vehicles: a classification and review of e-vehicle services. Transport. Res. D: Transport Environ..

[bib28] Morshed M.J., Hmida J.B., Fekih A. (2018). A probabilistic multi-objective approach for power flow optimization in hybrid wind-PV-PEV systems. Appl. Energy.

[bib29] Muratori M. (2018). Impact of uncoordinated plug-in electric vehicle charging on residential power demand. Nat. Energy.

[bib30] Raghavan S.S., Tal G. (2020). Plug-in hybrid electric vehicle observed utility factor: why the observed electrification performance differ from expectations. Int. J. Sustain. Transport..

[bib31] Rahbari O., Vafaeipour M., Omar N., Rosen M.A., Hegazy O., Timmermans J.M., Heibati S., Van Den Bossche P. (2017). An optimal versatile control approach for plug-in electric vehicles to integrate renewable energy sources and smart grids. Energy.

[bib32] Rezaei N., Khazali A., Mazidi M., Ahmadi A. (2020). Economic energy and reserve management of renewable-based microgrids in the presence of electric vehicle aggregators: a robust optimization approach. Energy.

[bib33] Sun B. (2021). A multi-objective optimization model for fast electric vehicle charging stations with wind, PV power and energy storage. J. Clean. Prod..

[bib34] Tal G., Raghavan S.S., Karanam V.C., Favetti M.P., Sutton K.M., Lee J.H., Nitta C., Chakraborty D., Nicholas M., Turrentine T. (2020). Advanced plug-in electric vehicle travel and charging behavior-final report. Calif. Air Resour. Board Contract.

[bib35] Taljegard M., Göransson L., Odenberger M., Johnsson F. (2019). Impacts of electric vehicles on the electricity generation portfolio – a Scandinavian-German case study. Appl. Energy.

[bib36] Thompson A.W., Perez Y. (2020). Vehicle-to-Everything (V2X) energy services, value streams, and regulatory policy implications. Energy Policy.

[bib37] Wu W., Lin B. (2021). Benefits of electric vehicles integrating into power grid. Energy.

