# Supplementary material for: Distribution grid impacts of electric vehicles: A California case study
Source: iScience. 2021 Dec 28;25(1):103686. doi: 10.1016/j.isci.2021.103686 (PMC8749456; doi:10.1016/j.isci.2021.103686)
Supplement: Document S1. Figures S1 and S2 [file mmc1.pdf]

iScience, Volume *25*

Supplemental information

Distribution grid impacts of electric vehicles: A California case study

Alan Jenn and Jake Highleyman

Supplemental Information

Supplemental Figures

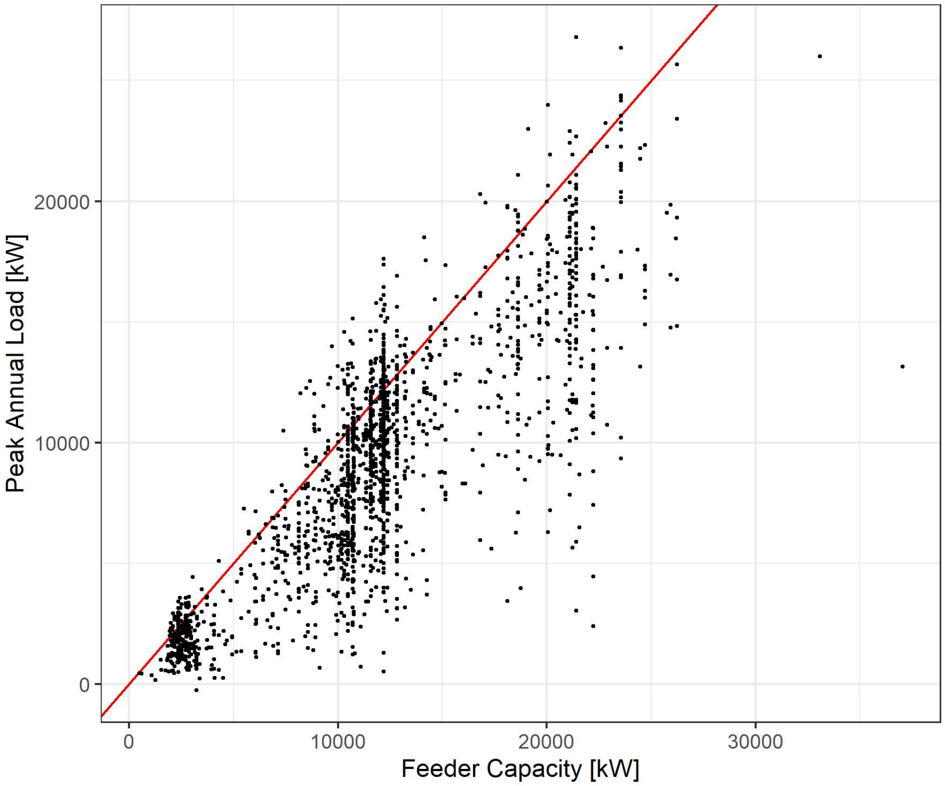

Figure S1: Feeder capacities versus the peak annual load observed on each corresponding feeder for all feeder circuits across PG&E's service territory. The red line indicates a match between capacity and peak load, points above this line represent feeders whose peak loads already exceed the capacity of the feeder at some point in the year. Related to STAR Methods.

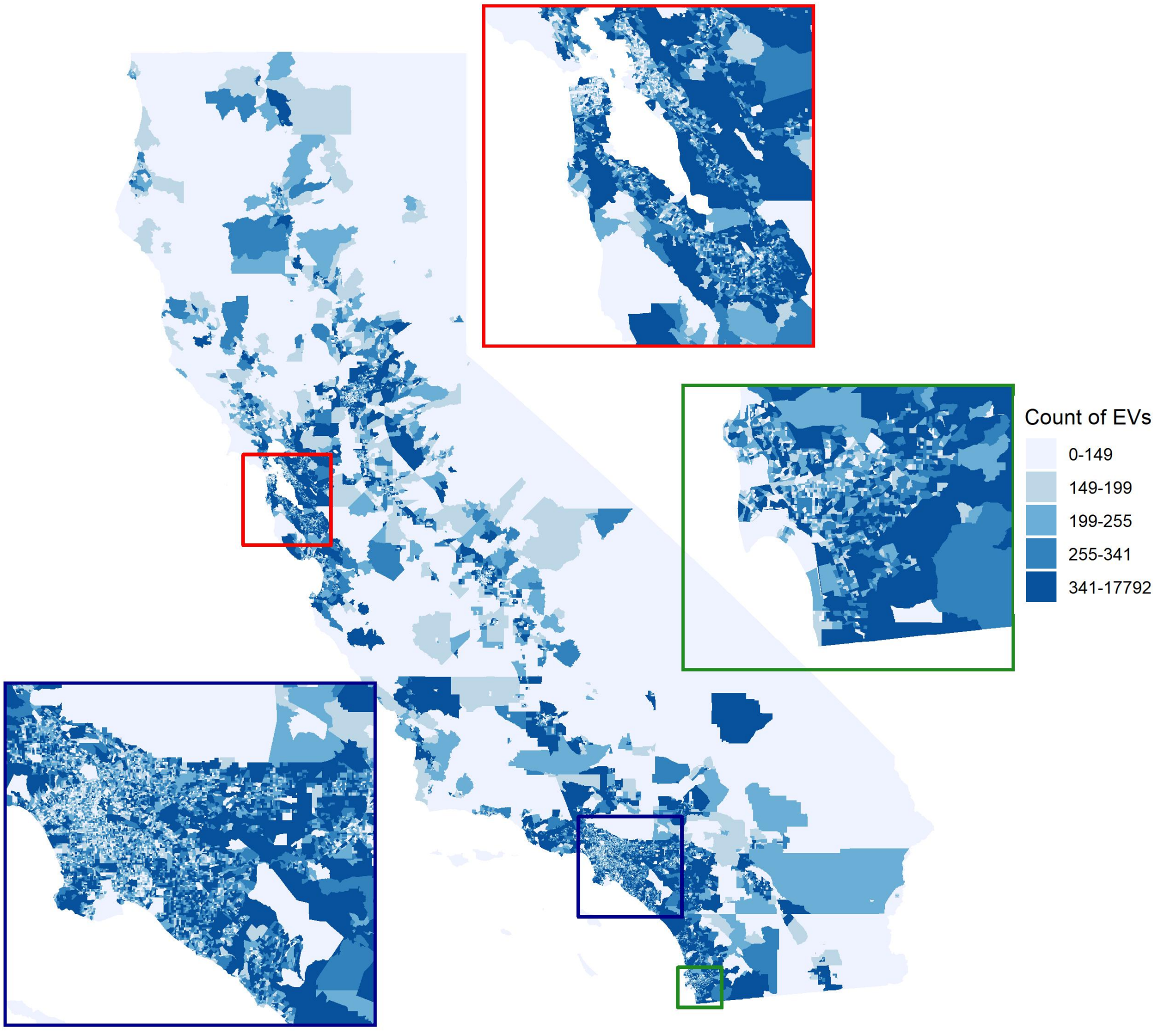

Figure S2: Sample output from EV Toolbox for 6 million EVs in California with a 75% BEV to 25% PHEV split. The EV adoption is provided at the census block group level. Related to STAR Methods.
